# Complete Genome Sequence of *Tessaracoccus* sp. Strain T2.5-30 Isolated from 139.5 Meters Deep on the Subsurface of the Iberian Pyritic Belt

**DOI:** 10.1128/genomeA.00238-17

**Published:** 2017-04-27

**Authors:** Tânia Leandro, Milton S. da Costa, Jose L. Sanz, Ricardo Amils

**Affiliations:** a Centro de Biología Molecular Severo Ochoa (CSIC-UAM), Universidad Autónoma de Madrid, Cantoblanco, Madrid, Spain; b Centre for Neuroscience and Cell Biology, University of Coimbra, Coimbra, Portugal; c Department of Life Sciences, University of Coimbra, Coimbra, Portugal; d Department of Molecular Biology, Universidad Autónoma de Madrid, Madrid, Spain

## Abstract

Here, we report the complete genome sequence of *Tessaracoccus* sp. strain T2.5-30, which consists of a chromosome with 3.2 Mbp, 70.4% G+C content, and 3,005 coding DNA sequences. The strain was isolated from a rock core retrieved at a depth of 139.5 m in the subsurface of the Iberian Pyritic Belt (Spain).

## GENOME ANNOUNCEMENT

The genus *Tessaracoccus* was described in 1999 and is classified within the family *Propionibacteriaceae*, phylum *Actinobacteria* (1). The genus contains nine validly published species, namely, *T. bendigoensis* (1), *T. flavescens* (2), *T. lubricantis* (3), *T. lapidicaptus* (4), *T. oleiagri* (5), *T. rhinocerotis* (6), *T. flavus* (7), *T. massiliensis* (8), *T. defluvii* (9), and one species, *T. profundi* (10), that has not been validly published. *Tessaracoccus* species have been isolated from diverse environments and are characterized as Gram-positive non-spore-forming facultative anaerobic bacteria (1). The type strains of *T. profundi* and *T. lapidicaptus* have also been isolated from deep subsurface environments (4, 10).

*Tessaracoccus* sp. strain T2.5-30 was isolated from a core sample from 139.5 m in the subsurface of the Iberian Pyritic Belt (IPB, Peña de Hierro, Spain). The IPB is characterized as one of the largest sulfide ore deposits known (11).

For whole-genome sequencing, DNA was extracted using a centyltrimethylammonium bromide (CTAB)-based extraction method (12). The quantity of extracted genomic DNA was determined with the Qubit version 2.0 fluorometer (Invitrogen, USA), and quality was analyzed by electrophoresis on an agarose gel, as well as on a NanoDrop 2000 (Thermo Scientific, USA) for measurement of the *A*_260_/*A*_280_ ratio. Genomic DNA was submitted to the Norwegian Sequencing Centre (University of Oslo, Norway) for PacBio single-molecule real-time (SMRT) sequencing (13). One SMRT cell was used for sequencing on a Pacific Biosciences RSII instrument using P6-C4 chemistry, with a 360-min movie time. The generated reads were then assembled using the Hierarchical Genome Assembly Process (HGAP) (SMRT Analysis software version 2.3.0; Pacific Biosciences) (14). The final assembly resulted in four contigs. The Minimus2 software (Amos package) was used to circularize the contigs (15). Circularization of contig 0 by joining and trimming of the overlapping 3′ and 5′ ends resulted in a circular closed chromosome. Contigs 1, 2, and 4 correspond to direct repeats of sequences contained in contig 0 and were excluded from our analysis.

The total genome length was approximately 3.2 Mbp, with a G+C content of 70.4%. The genome did not contain plasmids. PacBio SMRT sequencing resulted in approximately 175% coverage. The complete genome was annotated with NCBI Prokaryotic Genome Annotation Pipeline (PGAP) and with Rapid Annotations using Subsystems Technology (RAST), as incorporated in the PATRIC server (16, 17). RAST predicted a total of 3,005 DNA coding sequences, six rRNA genes, and 46 tRNA genes.

The comprehensive analysis of the complete genome sequence of *Tessaracoccus* sp. strain T2.5-30 will provide insights into the genetic potential of this strain to elucidate the mechanisms used by life to inhabit deep terrestrial environments in the absence of light as well as under oligotrophic conditions.

### Accession number(s).

The nucleotide sequence for the *Tessaracoccus* sp. strain T2.5-30 complete genome has been deposited at DDBJ/ENA/GenBank under the accession number CP019229. The version described in this paper is the first version.
